# Molecular profile of breast cancers in Guinean oncological settings

**DOI:** 10.11604/pamj.2019.33.22.18189

**Published:** 2019-05-14

**Authors:** Bangaly Traoré, Moussa Koulibaly, Aissatou Diallo, Malick Bah

**Affiliations:** 1 Surgical Oncology Unit, Donka National Hospital, Faculty of Medical Sciences and Technics, University Gamal Abdel Nasser of Conakry, Conakry, Guinea; 2 Laboratory of Anatomo-Pathology, Donka National Hospital, Faculty of Medical Sciences and Technics, University Gamal Abdel Nasser of Conakry, Conakry, Guinea; 3 Surgical Oncology Unit, Donka National Hospital, Faculty of Medical Sciences and Technics, University Gamal Abdel Nasser of Conakry, Conakry, Guinea

**Keywords:** Breast cancer, molecular subtypes, oncology

## Abstract

Breast cancer is a complex disease characterized by the accumulation of multiple molecular alterations giving each tumor phenotype and an own evolutionary potential. This study aimed to describe the distribution of the profile and molecular subtypes of breast cancers followed at Surgical Oncology Unit of Donka National Hospital. This was retrospective and descriptive study on cases of breast cancer in which the hormone receptor status and expression of the Her2 oncogene have been performed from 2007 to 2016. We recorded 58 cases including 56 (96.6%) women and 2 (3.4%) men. The average age was 48.2 ± 10.9. Invasive ductal carcinoma accounted for 50 (86.2%) cases. The SBR grade was II in 31(53.4%) cases, III in 21 (36.2%) cases and I in 6 (10.3%) cases. The tumor was classified as T4 in 36 (62.1%) cases; it was metastatic in 11(19.0%) cases. Estrogen receptors were positive in 29 (50.0%) cases, progesterone receptors positive in 25 (43.1%) cases, the Her2 oncogene was positive in 22 (39.3%) cases. The distribution of molecular sub-types was: 20 (34.5%) luminal A, 15 (25.9%) triple negative, 13 (22.4%) Her2 overexpressed, 8 (13.8%) luminal B and 2 (3.2%) undetermined. This preliminary study showed the poor accessibility of immunohistochemistry for the molecular diagnosis of breast cancer in our country. Luminal A subtypes and triple negatives were more common. The determination of molecular subtypes is a rational basis for hormone therapy and targeted therapy, thus personalizing the treatment of breast cancer.

## Introduction

Breast cancer is a major global public health problem. About 1,671,149 cases are diagnosed each year, including 521,907 deaths worldwide [1]. The number of cases reached 53,000 and more than 11,000 deaths in France [2]. In Africa, the incidence is 133,390 new cases with 63,160 deaths [1]. In North and Middle East Africa, breast cancer is the first cancer in women. It accounts for 14 to 42% of all female cancers with an exponential increase [3]. In Guinea, the age-standardized incidence is 14.5 new cases per 100,000 and the mortality rate is 7.9 per 100,000 [1]. It represents the leading cause of consultation at the Surgical Oncology Unit (SOU) of Donka National Hospital and represents 26% of all cancers [4]. In sub-Saharan countries, the patients are relatively younger and the stages are aggressive [5, 6]. Breast cancer now appears as a complex disease characterized by the accumulation of multiple molecular alterations that give each tumor a phenotype and a potential for evolution. The study of genomic alterations of tumor cells revealed their relation with the prognosis and the effectiveness of the treatments; thus requiring making a personalization in the therapeutic management. The early distinction of responder and non-responder patients is to avoid the use of ineffective chemotherapies for some patients. The progress of molecular biology techniques and gene sequencing allowed understanding the breast cancer genesis. Perou *et al.* [7] analyzed in 2000 the gene expression of breast cancer by micro-array and were able to highlight five molecular groups. This molecular classification distinguishes the luminal A, luminal B, Her2 positive, basal-like, and triple-negative subtypes [8]. Indeed, immunohistochemistry is used to define biological prognostic factors and especially to make a target therapy. This target therapy has been made possible through the use of monoclonal antibodies and hormone therapy. In our country, immunohistochemistry (IHC) is not available and there are very little data on the molecular profile of breast cancer [4, 9]. This study aimed to determine the first trends in the molecular profile of breast cancers followed at the surgical oncology unit of Donka National Hospital.

## Methods

This was retrospective and descriptive study on cases of breast cancer in which the hormone receptor status and expression of the Her2 oncogene have been reported. These patients were followed at the Surgical Oncology Unit (SOU) of the Donka National Hospital, University Hospital Center of Conakry, from 2007 to 2016. Age, sex, menopausal status for women and existence of comorbidity were recorded. The histological diagnosis of cancer was confirmed in the two pathology laboratories at Donka National Hospital and China-Guinean Kipe Hospital. The breast cancer characteristics were studied through the histological type, Scarff Bloom Richardson (SBR) grade, initial clinical classification and the Union for International Cancer Control (UICC) tumor, node and metastasis (TNM) stage. For immunohistochemistry, paraffin blocks were sent to the Cerba laboratory (Paris) via Dakar (Senegal) in the majority of cases. The status of estrogen (ER) and progesterone (PR) receptors and human epidermal receptor (Her2) oncogene expression were used to establish the classification of molecular subtypes of breast cancer (Table 1). The index ki-67, if available in the patient's file was used. The molecular subtype was considered indeterminate if any of the three elements (ER, PR, Her2) were not reported. We reviewed the indications of hormone therapy and targeted therapy (trastuzumab) according to the hormonal status and expression of the oncogene Her2. Statistical analysis was performed using SPSS software (SPSS Inc., Chicago, IL). Categorical variables were shown as the frequency and percentage (%) and continuous variables were presented as the mean and standard deviation (±SD). In this retrospective study, data were collected anonymously and confidentially. Patients signed the consent form for the use of data contained in their records.

**Table 1:** classification of molecular subtypes

Molecular subtypes	Criteria
Luminal A	ER (+), PR (+) et Her-2 (-)
Luminal B	ER (+), PR (+/-) et Her-2 (+)
Her-2	ER (-), PR (-) et Her-2 (+)
Triple negative	ER (-), PR (-) et Her-2 (-)

## Results

**Figure 1:**
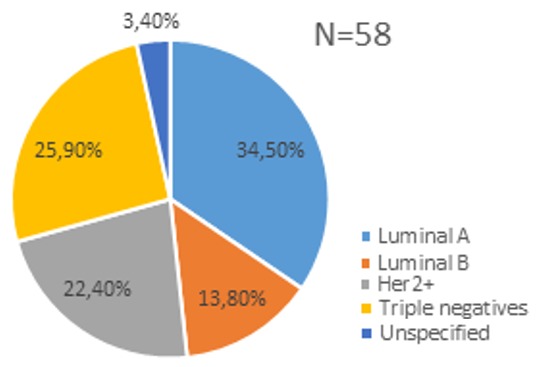
distribution of molecular subtypes

From 2007 to 2016, IHC performed in 58 patients (10.19%) out of a total of 569 breast cancers (Figure 1). The average age was 48.3 ± 10.9 years with extremes of 31 and 80 years. There were 56 women (96.6%) for 2 men (3.4%). There were 30 postmenopausal women (51.7%). Invasive ductal carcinoma was the most common histological type with 86.2%, an intra-ductal component was associated in 8 cases (13.8%). The Scarff Bloom and Richardson grades were SBR II in 31 cases (53.4%), SBR III in 21 cases (36.2%) and SBR I in 6 cases (10.3%). The tumor stage was less than or equal to T2 in 14 cases (24.1%), and greater than T2 in 44 cases (75.9%). There were axillary and/or subclavicular lymph nodes in 36 cases (62.1%). Locally advanced and metastatic stages were most common in 28 cases (48.3%) and 11 cases (19.0%). Table 2 presents the patients and breast cancer characteristics. Of the 58 cases, 29 (50.0%) had positive hormone receptors (HR+). ER were positive in 29 cases (50.0%) while PR were positive in 25 cases (43.1%). In 56 cases, the Her2 oncogene was positive in 22 cases (39.3%). The Ki67 index was known for 14 cases including 4 (6.8%) less than 15% and 10 (17.2%) greater than or equal to 15%. The distribution of the molecular sub-types was 34.5% luminal A, 25.9% triple negative, 22.4% Her2 + and 13.8% luminal B. The tumors of undetermined molecular profile were 3.4%. Chemotherapy was used in 94.8% of cases. Hormone therapy was used in 18 patients (72.0%). Only 4 (18.2%) patients with Her 2+ were treated with trastuzumab.

**Table 2:** patients and breast cancer characteristics

Characteristics	Number	%
Age (N=58)		
≤ 50 ys	32	55,2
**> 50 ys**	26	44,8
Sexe (N=58)		
Female	56	96,6
Male	2	3,4
Menopause (N=56)		
Yes	30	51,7
No	26	48,3
Histolgical type (n=58)		
Invasive ductal carcinoma	50	86,2
Invasive lobular carcinoma	2	3,4
Mucinous carcinoma	2	3,4
Carcinoma no other specified	5	5,1
Mixed carcinoma	1	1,7
SBR Grade (N=58)		
SBR I	6	10,3
SBR II	31	53,4
SBR III	21	36,2
**Primary tumor (N=58)**		
T1-T2	10	17,2
T3-T4	45	77,6
Tx	3	5,2
Regional lymph node		
Yes	51	87,9%
No	7	12,1%
Metastases (N=58)		
M0	47	81,0
M1	11	19,0
Clinical stage (N=55)		
Stage I-II	16	27,6
Stage III-IV	39	67,2
Unknown	3	5,2

## Discussion

This study highlights the dire need for IHC in sub-Saharan Africa. In this retrospective study, IHC was performed for 10.2% of breast cancers diagnosed from 2007 to 2016. Access to IHC remains limited, as it accounts for less than 30% of breast cancers in West African countries [10, 11]. It is often during study projects, whether monocentric [12] or multicenter [13], that the molecular profile is better performed in different sub-Saharan Africa countries. On the other hand, there is better access in the Maghreb countries [14]. IHC is not available in our country as in most countries of sub-Saharan Africa. For the cases in this study, the histological diagnosis is made in Conakry, the paraffin blocks are sent to other countries or when the patients stay there for treatment. Despite the small number of cases, this study has established the first trend of hormone receptor expression, Her2 oncogene and molecular sub-types of breast cancer in Guinea. Breast cancer is a complex and heterogeneous disease associated with clinicopathological and biological behaviors that vary from one population to another [15]. These prognostic factors are essential for the management of breast cancer. In addition, the molecular classification is now an important tool to guide breast cancer management. In this study, the average age was 48.2 years. This age is similar to that found in a previous study of all breast cancers at the SOU of Donka [5]. The relatively younger age of women with breast cancer is already reported in several studies in Africa [12, 16] unlike developed countries where the age is more advanced, beyond 65 [17]. Gakwaya *et al.* [18] in Uganda reported in their study a high frequency in the 30-39 age groups. Age at diagnosis is an important prognostic factor since tumors diagnosed at younger ages are generally more aggressive and/or less response to treatment. This young age is related to the age of the female population in Guinea, whose under-40s represent 83.1% [19]. The anatomo-clinical characteristics of breast cancer in this study are the same as those of breast cancer reported in a previous study [5]. They are characterized by tumors larger than T2 (75.9%), stage III and IV (70.9%), high grade (SBR II and III) (89.6%), and metastatic (19.0%).

Intensive Care Consortium (ICC) was the common histological type in more than 80% as in several other studies in Africa and elsewhere [14, 13, 20]. However, many efforts still have to be made by Guinean pathologists as to the description of histological types. In western countries, the majority of breast tumors are less than 2cm, reflecting early detection of the disease [21]. We did not take into account the tumor size in this study but 75.9% were classified T3-T4. These same proportions are found in multicentric studies in sub-Saharan Africa [22]. The advanced stage of diagnosis in our patients could be explained by the young age, the delay of consultation, the absence of mass screening in the population, the bad orientation and an insufficient reference system of the cancer patients. HRs were positive in half of the patients in our study. The positivity of HR in breast cancer remains very varied and heterogeneous in countries. The results are similar in South Africa [23] and Ghana [11]. HR positivity is very high in the Maghreb and the Western countries, possibly exceeding 70% [24]. These differences would be related to the young age of our patients even though patients with HR positive were relatively older than patients with HR negative. HR positivity has been shown to be greater in older patients than in the elderly [25]. HR is different depending on the types of receptors. In our study, ER were positive in 50.0% while PR were positive in 43.1%. This receptor positivity appeared to be higher than in Mali where Ly *et al.* [26] showed that ER and PR were positive in 39% and 29% respectively. In a multicentric study including 507 patients from Senegal and Nigeria, Huo *et al.* [27] demonstrated a low HR positivity rate, in the order of 24 and 20% for ER and PR. The HR status is the best indicator for hormone therapy. The small size of the samples published in different African countries makes it difficult to determine the factors that influence the expression of hormone receptors. This study justified tamoxifen hormone therapy in 72.0% of patients in our study who were RH positive.

Oncogene HER2 was positive in 39.3% of patients in our study. This expression is lower than that of hormone receptors. The Her2 oncogene is less positive, with a proportion of around 17% in Senegal and Nigeria [28]. Although being a target therapy, the overexpression of Her2 is associated with poor prognostic factors correlated to the stage of breast cancer diagnosis. Data showed that expression of the Her2 increased according to stage, 22% in the early stage, 35% in the locally advanced stage and 40% in inflammatory breast cancers [28, 29]. The distribution of molecular subtypes found a high frequency of luminal A subtypes (34.5%), followed by triple negatives (25.9%), Her2 + types (22.4%) and luminal B (13.8%). In sub-Saharan Africa, luminal A is more common in some countries [12, 30], while in others it is the triple negative that is more common [26, 27]. Apart from locoregional (by surgery and radiotherapy) and systemic (by chemotherapy) control, triple negative breast cancers are very difficult to manage in case of resistance to chemotherapy due to the lack of therapeutic target. The numbers are small, which could be used to highlight the lack of services in Guinea. The number of patients treated with hormone therapy (72.0%) and trastuzumab (18.2%) are small, which could be used to highlight the lack of services in Guinea.

## Conclusion

This preliminary study showed the poor accessibility of immunohistochemistry for the molecular diagnosis of breast cancer in our country. Luminal A subtypes and triple negatives were more common. The determination of molecular subtypes is a rational basis for hormone therapy and targeted therapy, thus personalizing the breast cancer treatment.

### What is known about this topic

- Breast cancer in sub-Saharan African women is aggressive: triple negatives and high grades.

### What this study adds

- We noted a difference in the distribution of molecular subtypes: luminal A more frequent than triple negative, but still high grade.
